# Predictors of mortality and disability in stroke-associated pneumonia

**DOI:** 10.1007/s13760-019-01148-w

**Published:** 2019-04-29

**Authors:** Rory J. Tinker, Craig J. Smith, Calvin Heal, Joao H. Bettencourt-Silva, Anthony K. Metcalf, John F. Potter, Phyo K. Myint

**Affiliations:** 1 Faculty of Biology, Medicine and Health, University of Manchester, Manchester, M13 9PL UK; 2 Greater Manchester Comprehensive Stroke Centre, Manchester Academic Health Science Centre, Salford Royal NHS Foundation Trust, Salford, M6 8HD UK; 3 Centre for Biostatistics, University of Manchester, Manchester Academic Health Science Centre, Oxford Road, Manchester, UK; 4 Room 4:013, Institute of Applied Health Sciences, School of Medicine, Medical Sciences and Nutrition, University of Aberdeen, Polwarth Building, Foresterhill, Aberdeen, AB25 2ZD Scotland, UK; 5 Norwich Medical School, University of East Anglia, Norwich, NR4 7TJ UK; 6 Stroke Research Group, Norwich Cardiovascular Research Group, Norwich Research Park, Norwich, NR4 7TJ UK; 7 Stroke Services, Norfolk and Norwich University Hospitals NHS Foundation Trust, Norwich, NR4 7UY UK

**Keywords:** Stroke, Pneumonia, Mortailty, Morbidity, Stroke associated

## Abstract

Whilst stroke-associated pneumonia (SAP) is common and associated with poor outcomes, less is known about the determinants of these adverse clinical outcomes in SAP. To identify the factors that influence mortality and morbidity in SAP. Data for patients with SAP (*n* = 854) were extracted from a regional Hospital Stroke Register in Norfolk, UK (2003–2015). SAP was defined as pneumonia occurring within 7 days of admission by the treating clinicians. Mutlivariable regression models were constructed to assess factors influencing survival and the level of disability at discharge using modified Rankin Scale [mRS]. Mean (SD) age was 83.0 (8.7) years and ischaemic stroke occurred in 727 (85.0%). Mortality was 19.0% at 30 days and 44.0% at 6 months. Stroke severity assessment using National Institutes of Health Stroke Scale was not recorded in the data set although Oxfordshire Community Stroke Project was Classification. In the multivariable analyses, 30-day mortality was independently associated with age (OR 1.04, 95% CI 1.01–1.07, *p* = 0.01), haemorrhagic stroke (2.27, 1.07–4.78, *p* = 0.03) and pre-stroke disability (mRS 4–5 v 0–1: 6.45, 3.12–13.35, *p* < 0.001). 6-month mortality was independently associated with age (< 0.001), pre-stroke disability (*p* < 0.001) and certain comorbidities, including the following: dementia (6.53, 4.73–9.03, *p* < 0.001), lung cancer (2.07, 1.14–3.77, *p* = 0.017) and previous transient ischemic attack (1.94, 1.12–3.36, *p* = 0.019). Disability defined by mRS at discharge was independently associated with age (1.10, 1.05–1.16, *p* < 0.001) and plasma C-reactive protein (1.02, 1.01–1.03, *p* = 0.012). We have identified non-modifiable determinants of poor prognosis in patients with SAP. Further studies are required to identify modifiable factors which may guide areas for intervention to improve the prognosis in SAP in these patients.

**Electronic supplementary material:**

The online version of this article (10.1007/s13760-019-01148-w) contains supplementary material, which is available to authorized users.

## Introduction

Stroke places a large burden on society causing approximately 11.8% of global deaths making it the second most common cause of death after ischaemic heart disease [1]. It is also the third most common cause of disability representing 4.5% of global Disability-Adjusted Life Years [2]. Pneumonia is a frequent clinical complication after stroke, occurring most often within the first week (stroke-associated pneumonia, SAP) [3–5]. It has been estimated that SAP complicates around 14% of strokes, although the frequency varies considerably between studies [5]. When SAP occurs, it has major clinical implications and has the highest attributable rise in mortality of all medical complications following stroke (estimated to be between 10.1 and 37.3%) [5]. Additionally, SAP has a large economic burden on medical systems with the cost of each case estimated to be £14,371 in the UK [6].

Currently the factors that determine mortality and morbidity in SAP are unknown [7]. This is in contrast to community-acquired pneumonia (CAP) where prognostic factors have been investigated leading to the development of prognostic scores (CURB-65) [8] which guide clinicians with regard to choice of antibiotics and route of administration. This has led to the care of pneumonia becoming more cost efficient, efficacious and personalised [9]. The use of CURB-65 has demonstrated that successful implementation of a risk stratification score in CAP can improve outcome, and a similar prognostic score in SAP could be of use in clinical practice and research [9]. The purpose of this study was, therefore, to investigate the factors that influence mortality and disability in SAP prior to development of a future prognostic score. The specific aim of the current study was to determine which patient-related factors were associated with mortality (at 1 and 6 months) and unfavourable functional outcomes in patients with SAP.

## Methods

We retrospectively analysed a prospectively collected, hospital-based stroke registry which included all consecutive stroke admissions to a regional stroke register. Ethical approval for the establishment of the database was granted by the Newcastle and Tyneside National Health Service (NHS) Research Ethics Committee (12/NE/0170).

### Data source

We selected eligible patients with confirmed stroke and SAP from the Norfolk and Norwich University Hospital (NNUH) Stroke Register. The register collects clinical information from consecutively admitted stroke patients to the tertiary centre in Norfolk, UK, which provides acute stroke services to a catchment population of 750,000 people. The cohort for the present study was constructed by extraction of anonymised electronic data of consecutive stroke admissions over 12 years (from January 2003 to April 2015) onto a secure central database. The data collection methods have been previously reported [10, 11]. In brief, acute stroke cases were diagnosed by the same firm of stroke physicians based on clinical features and imaging (CT/MRI). SAP was defined as pneumonia diagnosed within 7 days of admission. The diagnosis of pneumonia was made by clinicians as per British Thoracic Society (BTS) guidelines which require confirmation changes on chest radiography consistent with pneumonia [12].

### Data collection

The incidence of SAP and other prevalent comorbidities were retrieved from specific hospital electronic and/or paper patient records and coded in the 10th revision of the International Classification of Disease (ICD-10). The following patient baseline variables were available: age; sex; date of admission; date of discharge; discharge destination; Oxfordshire Community Stroke Project (OCSP) classification; type of stroke (haemorrhagic or ischaemic stroke); length of hospital stay (LOS); pre-stroke modified Rankin Scale (mRs) (6-point score 0–5); post stroke discharge mRS (7-point score 0–6 incorporating death as score 6) and antiplatelet treatment (at admission). The dataset also included routine blood tests. Blood tests results were included in the analysis if they were taken within ± 5 days of the diagnosis of SAP. The specific haematological and biochemical variables available for investigation were as follows: urea and electrolytes (Us and Es); full blood counts (FBC); International Normalized Ratio (INR), thyroid function tests (TFTs) and acute inflammatory response markers (total white cell count (WCC) and C reactive protein (CRP). Comorbidities included previous: stroke; transient ischaemic attack (TIA); coronary heart disease (CHD); myocardial infarction (MI); atrial fibrillation; congestive heart failure (CHF), hypertension; dyslipidaemia; diabetes mellitus; peripheral vascular disease (PVD); chronic obstructive pulmonary disease (COPD); asthma; previous pneumonia; chronic kidney disease (CKD); malignancy; vascular dementia; and Alzheimer’s disease. The length of survival after stroke was derived from time between the patient’s admission to hospital and the date of their death (if applicable) or censor date (30 or 90 days post admission). Electronic linkage with the Office of National Statistics (ONS) for mortality data enables complete follow up for mortality.

### Statistical analysis

SPSS for Windows version 23.0 (SPSS Inc., Chicago, IL, USA) was used to perform the statistical analysis. The primary outcomes of interest were mortality at 1 month and at 6 months from admission to hospital for the index stroke. The secondary outcomes were poor functional outcome defined as mRs score at discharge (0–4 = good, 5 + = bad), dichotomised based on the median discharge mRS.

Univariable logistic binary regression models were used to investigate the associations between discrete categorical variables and mortality. Univariable linear regression models were constructed to examine the associations between continuous variables and mortality. Following this, variables were inputted into multivariable logistic regression models based on *p* value threshold of < 0.10 (10% significance level) in the univariable analyses, clinical experience, and the literature on stroke and pneumonia prognostic factors. The dataset for mortality was analysed twice. The first analysis was restricted to variables with complete data sets with no missing variables (all baseline characteristics, including comorbidities) (analysis 1). As there were missing values for blood tests and pre stroke mRS, in the second round of analysis all variables were analysed regardless of completeness of data set. This leads to the introduction of the blood results and pre stroke modified rankin scores into the analysis (analysis 2).

To further investigate the effect of age and pre stroke disability, the cohort was stratified into nine distinct groups based on increasing age and pre stroke disability. All variables were analysed regardless of data completeness. These strata were then analysed in multivariable logistic regression models (correcting for factors influencing disability in the previous multivariable models) at both 1 and 6 months. This resulted in correction for age and the following commodities: dementia, lung cancer and TIA at 6 months. At 1 month this resulted in correction for age, the type of stroke (haemorrhagic) and pre stroke disability.

## Results

854 patients with SAP were included in the final analysis from the cohort of 11,886 in the NNUH register. The baseline characteristics are summarised in Table 1. Overall, the median (IQR) age was 83 year (77.5–88.5), 49% were male and the majority of patients presented with ischaemic stroke (85.1%).

**Table 1:** Baseline characteristics of participating patients

	Cohort with SAP
*n*	854
Male (%)	419 (49.1)
Age group, *n* (%)	
< 60	15 (1.8)
60–69	59 (7.2)
70–79	220 (27.0)
80–89	403 (49.5)
90 +	158 (19.4)
Median age (IQR) (years)	83 (77.5–88.5)
Age standard deviation (years)	8.749
Stroke type, *n* (%)	
Ischemic	727 (85.1)
Haemorrhagic	127 (14.9)
Vascular risk factors	
Hypertension, *n* (%)	324 (37.9)
Atrial fibrillation	241 (28.2)
Diabetes, *n* (%)	102 (11.9)
Mortality	
7-day mortality, *n* (%)	36 (4.2)
30-day mortality, *n* (%)	164 (19.2)
6-month mortality, *n* (%)	378 (44.2)
Final mortality, *n* (%)	523 (61.2)

In the univariable regression models, 30-day mortality (Table 1 of Supplement) was associated with the following: increasing age (for each additional life year), stroke subtype (haemorrhagic) and the following comorbidities: dementia, lung cancer. In the univariate regression models mortality at 6 months (Table 2 of Supplement) was associated with increasing age (for each additional life year), stroke subtype (haemorrhagic) and the following comorbidities: dementia, lung cancer.

In the univariable analyses, disability at discharge was predicted by increasing age and serum C-reactive protein levels (Table 3 of Supplement).

When analysis was conducted with only complete data sets with no missing variables (analysis 1, Table 2) mortality at 30 days was predicted by the following: a patient’s increasing age (for each additional life year), the type of stroke (haemorrhagic) and the following commodities: dementia, lung cancer.

**Table 2:** Factors associated with mortality and discharge disability in the multifactorial analysis

		30-day mortality		6-month mortality		Discharge disability
		Complete only	All	Complete only	All	–
Independent variable	*n*	OR (95% CI), *p*	OR (95% CI), *p*	OR (95% CI), *p*	OR (95% CI), *p*	OR (95% CI), *p*
Age (per 1-year increase)	854	1.04 (1.01, 1.06), 0.005	1.04 (1.01, 1.07), 0.011	1.07 (1.05, 1.09, < 0.001)	1.08 (1.05, 1.12), < 0.001	1.10 (1.05–1.16), < 0.001
Haemorrhagic stroke	854	2.49 (1.26, 4.92), 0.009	2.49 (1.26, 4.92), 0.032			–
Comorbidities	–	–	–	–	–	–
All dementia	854	12.90 (7.50–22.20), < 0.001	–	6.53 (4.73,9.03), < 0.001		–
Lung cancer	854	8.91 (2.70–29.38), 0.001	–	2.07 (1.14, 3.77), 0.017		–
TIA	854	–	–	1.94 (1.12, 3.36)	–	–
Previous pneumonia	854	–	–	–	1.77 (1.10, 2.85), 0.020	–
Pre-stroke disability	–	–	–	–	–	–
0–1	220	–	1	–	1	–
2–3	101	–	4.65 (2.36, 9.15), < 0.001	–	2.06 (1.21, 3.51), 0.008	
4–5	94	–	6.45 (3.12, 13.35), < 0.001	–	3.00 (1.54, 5.84), 0.001	
C-reactive protein (mg/dl)		–	–	–	–	1.02 (1.01–1.03), 0.012

When all available data were analysed (regardless if the data set had missing variables or not) (analysis 2 Table 2), multivariable regression models predicted mortality at 30 days based on the following variables increasing age (for each additional year of life year), stroke subtype (haemorrhagic) and pre stroke modified rankin.

Multivariable regression models which included only the complete data sets with no missing values (analysis 1, Table 2) predicted mortality at 6 months bythe following: a patient’s increasing age (for each additional life year) and the following comorbidities: dementia, lung cancer and TIA.

When all available data were analysed (regardless if the data set had missing variables or not) (analysis 2 Table 2) mortality was predicted at 6 months by: a patients’ increasing age (per a year), pre stroke disability, and a previous episode of pneumonia. The inclusion of pre stroke modified Rankin, therefore, removed comorbidities (dementia and lung cancer). All multivariable models for mortality are displayed in Table 1.

In the multivariable logistic regression model, disability at discharge was predicted by increasing age (per year) and serum C-reactive protein levels (Table 2).

**Fig. 1:**
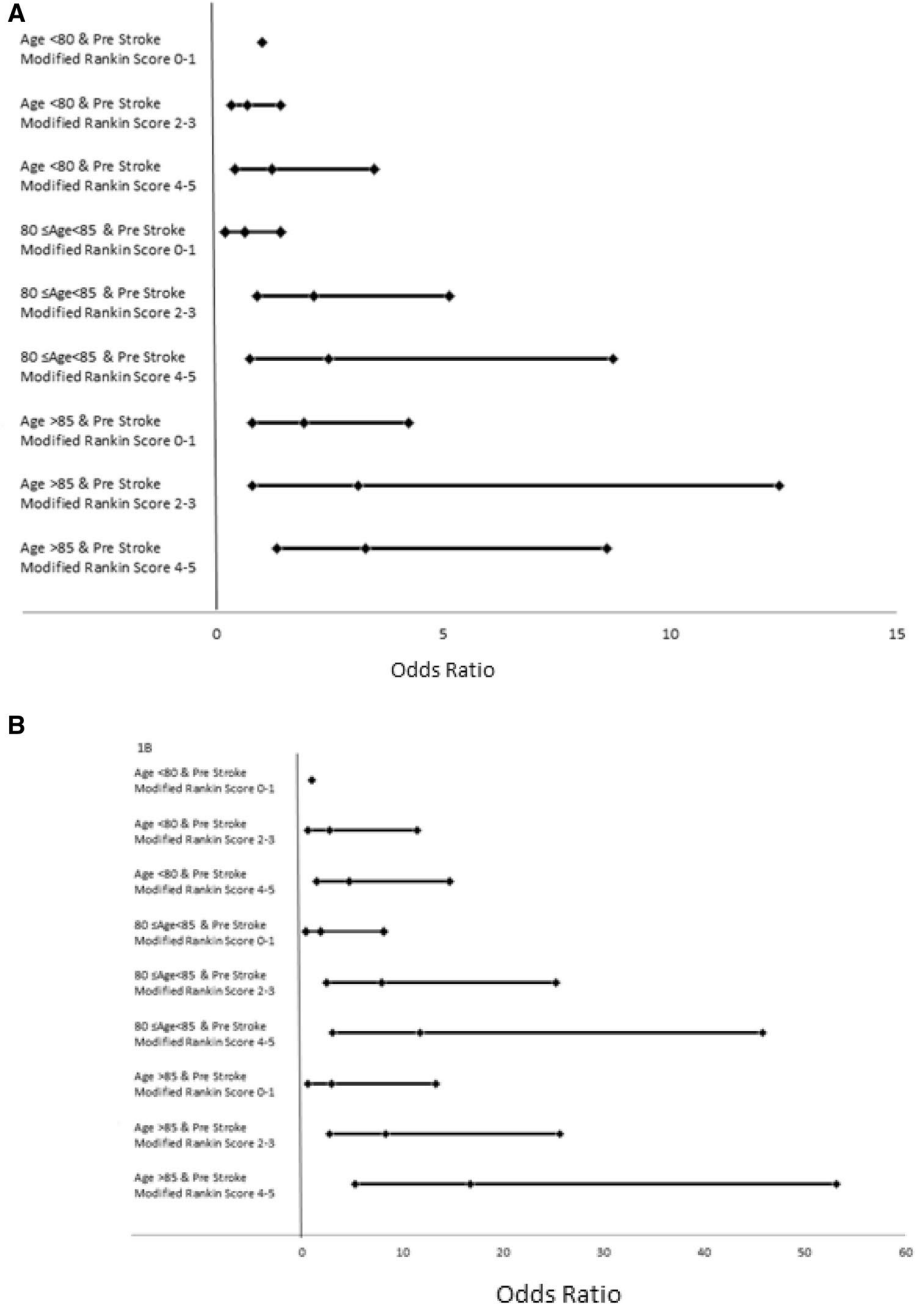
Forest plot showing effect of stratification of age and pre-stroke mRS on, **a** mortality at 30 days and **b** mortality at 6 months. Each data point represents OR with 95% CI

When the cohort was split into 9 strata for age and pre-stroke disability, mortality at both 1 and 6 month was associated with being in an older age group and being in a more disabled Rankin group (Fig. 1; Table 4 of Supplement). Age and disability were of greater predictive value at 30 days.

## Discussion

SAP has a major impact on morbidity and mortality following stroke [10]. As the prognostic factors are currently unknown, we aimed to explore clinical characteristics predictive of poor outcome in SAP. The major findings of the study are that mortality is associated with non-modifiable patient characteristics including increasing age, haemorrhagic stroke sub-type, pre-stroke disability and comorbidities. An additional finding of the study was that disability after the stroke was predicted by age and serum CRP.

The characteristics of the patients who developed SAP is similar to previously reported literature. The patients tended to be older, frailer and had higher number of comorbidities [9, 11]. This is in keeping with previous analysis of the full cohort from which the SAP sample was extracted that has shown those patients who develop SAP after stroke older, more frail and more comorbid then the patients who do not [9].

When the results of this study are compared to the literature for CAP which is usually caused by viral or bacterial infection there appear to be some overlap. Age predicts survival at 1 month and 6 months in CAP in the literature and for SAP the current study [8, 13–15]. When comparing to the CURB-65 score for CAP, we did not find an association between serum urea concentrations at diagnosis of SAP and mortality [9]. It is difficult to conclude if this is a true finding or due to the incomplete data for urea concentration (sees limitations of study) and therefore the current study cannot exclude such an association and this should be investigated in the future studies of SAP.

Prestroke mRS has been shown to be a risk factor for the development of pneumonia after stroke, but the finding that it is associated with death after SAP has not been reported to our knowledge [7]. The results of this study are in keeping with the general literature on stroke prognosis [16–18]. The current study also suggests that age and disability are of greater prediction for mortality in the first month after the stroke than at 6 months suggesting they are both of greater utility acutely than long term.

To the best of our knowledge, the finding that age and peripheral inflammatory state affect disability after SAP has not been reported previously [19]. In addition our findings are in keeping with the general literature with regard to disability after stroke [18, 19]. The literature suggests that older patients are more likely to have a higher level of disability post stroke (like SAP) [20]. Additional inflammatory markers (including CRP) have been associated with poor outcome after stroke and greater disability [18]. Also, plasma CRP detects early development of stroke-associated infections and may have a role in diagnostic criteria for SAP [14]. It is therefore likely that the association with CRP could be due to more severe cases of SAP and higher stroke severity (which are more likely to cause disability) leading to higher levels of inflammatory makers [21]. This could therefore mean that CRP represents both a diagnostic marker for SAP and also the severity and prognosis of SAP. Identification of increase CRP as a poor prognostic marker in SAP provides an intriguing possibility that the attempt to reduce inflammation and possible associated infection may reduce the excess mortality and morbidity burden of SAP.

The current study has some limitations. Firstly, it is based on secondary analyses of a single prospective cohort from a geographically defined part of the UK. We have various missing values limiting the multiple regression models that were subsequently created. However, this should not have impact on internal association between predictors and outcomes. Furthermore, the study was based on the retrospective analysis of digitalised patient records. This meant that the timings of blood results inputted into the study did not always correspond exactly to the diagnosis of SAP further limiting the regression model. The data also lacked basic physiological and functional data measurements (oxygenation, blood pressure, respiratory rate, dysphgia status) and cognition (e.g. MMSE) as well as information on stroke severity (e.g. NIHSS scores) [22]. Many of these variables have been associated with survival post stroke or CAP and therefore have the potential to be associated with survival post SAP and potential confounders to the relationship between outcome and the variables we found to be associated [23, 24]. For example, NIHSS is independently associated with both morbidity and mortality post-stroke as well the risk of developing SAP. However, we were able to control for Oxfordshire Community Stroke Project classification as indicator of stroke severity. Additionally in some cases, clinical decision may be made to withdraw treatment at the time of SAP diagnosis and this may have impact. As a large real world observational study, we are not able to address in this work.

The strengths of the study include employing a consistent and reproducible data collection at a single centre from the same groups of clinicians and their team making data collection accurate, reproducible. Secondly the study sample size was large and comprehensive covering many of the large number comorbidities which has been shown to have prognostic value in stroke.

This study describes factors influencing mortality and disability after SAP which are both largely consistent with the literature for both pneumonia and stroke. To expand on these findings it would be helpful to conduct a prospective study on the prognostic factors for SAP on different cohorts. Blood tests and physiological observations could then be taken at the specific time of diagnosis with SAP.

Finally in order to make this data clinically applicable further research is required to develop the findings into a prognostic score. Such a score could be used at the bedside to identify stroke patients at greatest risk of SAP creating the possibility to personalise their treatment and ultimately reduce mortality.

## Electronic supplementary material

Below is the link to the electronic supplementary material.

Supplementary material 1 (DOCX 30 kb)
