# Supplementary material for: Predictors of mortality and disability in stroke-associated pneumonia
Source: Acta Neurol Belg. 2019 Apr 29;121(2):379–85. doi: 10.1007/s13760-019-01148-w (PMC7956938; doi:10.1007/s13760-019-01148-w)
Supplement: Supplementary file 1 — Supplementary material 1 (DOCX 30 kb) [file 13760_2019_1148_MOESM1_ESM.docx]

Supplemental Tables:

Sup Table 1: Factors associated with 30 day mortality in univariable analyses

Sup Table 2: Factors associated with 6 month mortality in univariable analyses

Sup Table 3: Factors associated with discharge disability in univariable analyses

Sup Table 4: Effect of stratification of age and pre stroke disability on mortality and discharge disability

Supplementary Table 1

| Independent variable | N= | Odds Ratio (95% confidence limits) | Significance |
| --- | --- | --- | --- |
| Age  (per 1-year increase) | 854 | 1.04 (1.05,1.06) | 0.002 |
| Age  (greater than Median (83)) | 854 | 1.99 (1.38, 2.88) | <0.001 |
| Male Sex | 854 | 1.05 (0.74, 1.49) | 0.778 |
| Type of Stroke | - |  |  |
| Haemorrhagic stroke | 854 | 3.21 (1.59, 6.48) | 0.001 |
| Pre Stroke Disability  (Pre Stroke Modified Rankin scale grouped) | 415 |  |  |
| 0-1 (1) | 220 | - | - |
| 2-3 (2) | 101 | 4.80 (2.36,9.77) | <0.001 |
| 4-5 (3) | 94 | 7.31 (3.41,15.69) | <0.001 |
| Comorbidity |  |  |  |
| Asthma | 854 | 0.88 (0.50,1.54) | 0.654 |
| Cancer (All) | 854 | 1.40 (0.89, 2.19) | 0.114 |
| Cancer (Lung) | 854 | 9.85 (1.35, 72.11) | 0.024 |
| Chronic kidney disease | 854 | 1.63 (0.75, 1.81) | 0.50 |
| Dementia (Vascular) | 854 | 18.16 (7.35,44.84) | <0.001 |
| Dementia (Other) | 854 | 28.34 (6.93,115.07) | <0.001 |
| Diabetes | 854 | 0.71 (0.38,1.325) | 0.285 |
| Emphysema | 854 | 1.70 (0.86,3.37) | 0.129 |
| Heart failure | 854 | 1.55 (1.01,2.39) | 0.047 |
| Hypertension | 854 | 1.55 (2.37,4.91) | 0.001 |
| Hyperlipidaemia | 854 | 2.96 (1.78,4.92) | 0.001 |
| Peripheral vascular disease (PVD) | 854 | 1.48 (0.79,2.79) | .226 |
| Previous Lower respiratory tract infection | 854 | 1.38 (1.02,2.89) | 0.043 |
| Previous Myocardial Infarction | 854 | 1.96 (0.85,2.25) | 0.196 |
| Previous Transient Ischemic attack | 854 | 1.96 (0.96,4.00) | 0.065 |
| Blood Tests | - |  |  |
| Albumin | - |  |  |
| Hypoalbuminemia (Albumin <3.5g/dL) | 344 | 0.95 (0.51,1.77) | 0.870 |
| C Reactive Proteins (>30mg/dl) | 351 | 1.41 (0.75,2.68) | 0.286 |
| Urea and Electrolytes |  |  |  |
| Hypernatremia  (Na+>145mmol/l+) | 305 | 13.61 (1.69,7.14) | 0.001 |
| Hyponatremia (Na+<135mmol/l+) | 305 | 1.21 (0.46,3.15) | 0.699 |
| Urea >9mmol/l | 446 | 4.64 (2.94,7.32) | <0.001 |

Supplementary Table 1. Univariable predictors of mortality at 1 month in Stroke associated pneumonia cohort.

Supplementary Table 2

| Independent variable | N= | Odds Ratio (95% confidence limits) | Significance |  |  |  |
| --- | --- | --- | --- | --- | --- | --- |
| Age  (per 1-year increase) | 854 | 1.06 (1.04,1.08) | <0.001 |  |  |  |
| Age  (greater than Median (83)) | 854 | 2.32 (1.76,3.07) | <0.001 |  |  |  |
| Male Sex | 854 | 0.95 (0.73,1.25) | 0.73 |  |  |  |
| Type of Stroke | - | - | - |  |  |  |
| Haemorrhagic stroke | 854 | 1.35 (0.92,2.00) | 0.006 |  |  |  |
| Pre Stroke Disability  (Pre Stroke Modified Rankin scale grouped) | 415 |  |  |  |  |  |
| 0-1 (1) | 220 | - | - |  |  |  |
| 2-3 (2) | 101 | 1.59 (0.97,2.61) | 0.07 |  |  |  |
| 4-5 (3) | 94 | 2.82 (1.50-5.3) | 0.001 |  |  |  |
| Comorbidity |  |  |  |  |  |  |
| Asthma | 854 | 1.13(0.78,1.64) | 0.52 |  |  |  |
| Cancer (All) | 854 | 0.823 (0.61,1.12) | 0.21 |  |  |  |
| Cancer (Lung) | 854 | 4.09 (2.576.51) | <0.001 |  |  |  |
| Chronic kidney disease | 854 | 1.25 (0.87,1.79_ | 0.01 |  |  |  |
| Chronic Obstructive Pulmonary Disease | 854 | 1.67 (1.25,2.25) | <0.001 |  |  |  |
| Dementia (All types) | 854 | 6.29 (4.64,8.53) | <0.001 |  |  |  |
| Dementia (Alzheimer’s) | 854 | 4.75 (3.48,6.47) | <0.001 |  |  |  |
| Dementia (Vascular) | 854 | 5.20 (3.04,6.61) | <0.001 |  |  |  |
| Dementia (Other) | 854 | 4.48 (2.14,5.27) | <0.001 |  |  |  |
| Diabetes | 854 | 1.25 (0.74,1.75) | 0.41 |  |  |  |
| Emphysema | 854 | 1.84 (1.21,2.80) | 0.004 |  |  |  |
| Heart failure | 854 | 1.45 (1.03,2.05) | <0.001 |  |  |  |
| Previous Lower respiratory tract infection | 854 | 1.64 (1.13,2.38) | 0.009 |  |  |  |
| Previous Myocardial Infarction | 854 | 1.06 (0.75,1.48) | 0.75 |  |  |  |
| Previous Pneumonia | 854 | 2.09 (1.32 ,3.30) | 0.004 |  |  |  |
| Previous Transient Ischemic attack | 854 | 2.33 (1.42,3.83) | <0.001 |  |  |  |
| Blood Tests | - |  | - |  |  |  |
| Albumin | - |  | - |  |  |  |
| Hypoalbuminemia (Albumin <3.5g/dL) | 344 | 2.24 (1.42,3.55) | <0.001 |  |  |  |
| C Reactive Proteins (>30mg/dl) | 351 | 2.29 (1.45,3.61) | <0.001 |  |  |  |
| Urea and Electrolytes |  |  |  |  |  |  |
| Hypernatremia  (Na+>145mmol/l+) | 305 | 6.23 (3.31,11.72) | 0.003 |  |  |  |
| Hyponatremia (Na+<135mmol/l+) | 305 | 1.01 (0.56,1.83) | 0.97 |  |  |  |
| Urea >9mmol/l | 446 | 3.32 (2.21,4.99) | <0.001 |  |  |  |

Supplementary Table 2. Univariable predictors of mortality at 6 months in Stroke associated pneumonia cohort.

Supplementary Table 3

| Independent variable | N= | Odds Ratio (95% confidence limits) | Significance |  |  |
| --- | --- | --- | --- | --- | --- |
| Age (per 1-year increase) | 320 | 1.04 (1.02,1.09) | <0.001 |  |  |
| Male Sex | 320 | 1.68 (0.73,3.87) | 0.22 |  |  |
| Pre Stroke Disability | 298 | 1.85 (1.18,2.80) | 0.008 |  |  |
| Co-morbidities |  |  |  |  |  |
| Asthma | 320 | 2.79 (1.03,7.53) | 0.043 |  |  |
| Cancer | 320 | 0.52 (0.17,1.56) | 0.25 |  |  |
| Cancer (Lung) | 320 | 0.5 (0.07-4.22) | 0.56 |  |  |
| Chronic kidney disease | 320 | 1.47 (0.41-5.27) | 0.55 |  |  |
| Chronic Obstructive Pulmonary Disease | 320 | 0.44(0.10-1.91) | 0.27 |  |  |
| Diabetes | 320 | 2.16 (0.91 ,5.12) | 0.08 |  |  |
| Emphysema | 320 | 0.64 (0.08,5.01) | 0.67 |  |  |
| Heart failure | 320 | 2.250 (0.95-5.344) | 0.07 |  |  |
| Previous Lower respiratory tract infection | 320 | 1.12 (0.32,3.95) | 0.86 |  |  |
| Blood Tests |  |  |  |  |  |
| Albumin |  |  |  |  |  |
| Hypoalbuminemia Albumin <3.5g/dL | 217 | 1.87 (0.66,5.27) | 0.24 |  |  |
| CRP (mg/dl)  Linear | 213 | 1.02 (1.01-1.04) | 0.003 |  |  |
| Urea >9mmol/l | 229 | 0.42 (0.14-1.33) | 0.141 |  |  |

Supplementary Table 3. Univariable predictors of post stroke disability (defined as modified Rankin score 4+) in Stroke associated pneumonia cohort.

| Multivariable at 6M | | Pre Stroke Modified Rank Score (OR,95%CL) | | |
| --- | --- | --- | --- | --- |
|  |  | 0-1 | 2-3 | 4-5 |
| Age | 80> | 1 | 0.68 (0.32,1.42) | 1.21 (0.42 ,3.49) |
|  | 80 ≤x<85 | 0.62 (0.20,1.40) | 2.14 (0.89,5.15) | 2.48 (0.74,8.74) |
|  | 85 ≤ | 1.92 (0.79,4.25) | 3.13 (0.79,12.43) | 3.38 (1.32,8.62) |
| Multivariable analysis at 1M | |  |  |  |
| Age | 80> | 1 | 2.74 (0.66,11.47) | 4.74 (1.53,14.70) |
|  | 80 ≤x<85 | 1.86 (0.43 ,8.16) | 7.95 (2.50,25.24) | 11.75 (3.01,45.84) |
|  | 85 ≤ | 2.95 (0.65,13.37) | 8.37 (2.73,25.69) | 16.73 (5.27,53.20) |

Supplementary Table 4. The Combined effect age and disability (assed through Pre Stroke Modified Rank Score) groups have on the risk of death at 1 and 6 months.
